# A17 EVIDENCE OF PROTEASE-MEDIATED PRO-NOCICEPTIVE EFFECTS OF FECAL SUPERNATANTS FROM CROHN'S DISEASE PATIENTS

**DOI:** 10.1093/jcag/gwac036.017

**Published:** 2023-03-07

**Authors:** H Wood, T Alward, A Abu Omar, M Guzman-Rodriguez, S Vanner, D Reed, A Lomax

**Affiliations:** Gastrointestinal Diseases Research Unit, Queen's University, Kingston, Canada

## Abstract

**Background:**

Abdominal pain is a debilitating symptom of Crohn’s disease (CD). Despite the current treatment options for this disease, abdominal pain is an unresolved problem that commonly persists in the absence of active inflammation. This suggests that something other than inflammation is driving the pain during the quiescent phase. We have previously reported that microbial proteases can directly modulate the excitability of dorsal root ganglia (DRG) neurons, many of which are pain-sensing. We hypothesize that luminal proteases of CD patients are contributing to their abdominal pain.

**Purpose:**

Determine whether luminal mediators in CD fecal samples induce changes in pain signalling.

**Method:**

The effects of patient (active CD [n = 3] and healthy volunteer (HV) [n = 3]) fecal supernatant (FS) samples on pain-sensing neurons were assessed using *ex-vivo* single unit afferent nerve recordings from mouse colons. Each sample was tested in colonic preparations from a least 5 mice. To further examine cellular mechanisms, DRG neurons were isolated and incubated overnight in media containing CD FS or HV FS media. Changes in neuronal excitability were recorded by determining the rheobase (lower rheobase=increased excitability) using patch clamp recordings (n ≥ 9 DRG neurons/group). Protease inhibitors were applied in both bioassays to determine whether these inhibited the excitatory effect of FS. Lastly, total proteolytic activity in the CD and HV fecal samples was calculated using a casein colorimetric protease detection assay.

**Result(s):**

FS from HV had no effect on afferent nerve excitability (p = 0.8920). FS from active CD patients increased action potential discharge from colonic afferent nerves by 85% (p<0.0001) and selectively increased the activation of high-threshold units, which are putative nociceptors, by 44% (p=0.0074). A protease inhibitor cocktail (1:1000) and protease-activated receptor (PAR)-2 antagonist GB83 (10µM) both blocked the excitatory effects of CD FS (p<0.05). Overnight incubation with CD FS also had an excitatory effect on DRG neurons compared to HV FS (rheobase decreased by 46%, p<0.05). The effect of CD FS was blocked by GB83 (10µM) (p<0.001) and a serine protease inhibitor (FUT175; 100µM) (p<0.05) independently, but the activity was not blocked by E64 (30nM) a cysteine protease inhibitor. A 200-fold increase (p<0.0001) in total proteolytic activity was found in CD FS compared to HV FS.

**Conclusion(s):**

Luminal serine proteases, but not cysteine proteases, appear to be driving nociceptive signalling in CD patients. This provides insight into the generation of pain in CD patients and may be a potential target to mitigate this action. Further research is required to elucidate whether these pro-nociceptive proteases are of bacterial or host origin and their effects in the quiescent phase.

**Disclosure of Interest:**

None Declared

